# Microplastics Removal by Sustainable PVA/Bentonite Membranes: Morphological and Structural Evidence of the Retention Mechanism

**DOI:** 10.1021/acsomega.6c02323

**Published:** 2026-06-11

**Authors:** Fernando Silva Dal Magro, Wendel Paulo Silvestre, Camila Baldasso

**Affiliations:** † Postgraduate Program in Process Engineering and Technologies (PGEPROTEC), 58802 University of Caxias do Sul, Street Francisco Getúlio Vargas, 1130, Petrópolis, Caxias do Sul, RS 95070-560, Brazil

## Abstract

The increasing presence of microplastics (MPs) in aqueous matrices has driven the search for efficient and sustainable technologies for their removal. In this context, this work investigates the development of green polymeric membranes based on poly­(vinyl alcohol) (PVA) reinforced with bentonite clay nanoparticles (ArNPben), aiming to establish relationships between structure, properties, and performance in microplastic filtration. The membranes were produced by the solvent evaporation phase inversion method, employing PVA at different concentrations (6–10% w/v), citric acid as a cross-linking agent, and glycerol as a plasticizer, with the incorporation of bentonite (1–5% w/w). Characterization was performed using morphological, spectroscopic, and thermal techniques (SEM, FTIR, TGA, and DSC), associated with the evaluation of transport behavior and retention efficiency. The results demonstrated that the controlled incorporation of bentonite promotes structural reorganization of the polymer matrix, directly affecting hydraulic permeability, stability, and interaction with particles. A critical dispersion limit of the inorganic phase was observed, where low concentrations favor the formation of homogeneous and functionally efficient structures, while higher concentrations induce structural heterogeneity and increased resistance to transport. The formulation containing 6% (w/v) PVA and 1% (w/w) bentonite showed the best overall performance, combining structural stability and permeation capacity, achieving an average PM removal efficiency of 99.78 ± 0.13% in tests conducted under controlled pressure. Evidence obtained by FTIR and TGA confirmed the retention of polymeric material on the surface of the membranes after use. The results highlight the potential of the developed hybrid membranes as a sustainable and technically viable alternative for application in water treatment systems contaminated by microplastics, contributing to the advancement of technologies based on materials with low environmental impact.

## Graphical abstract

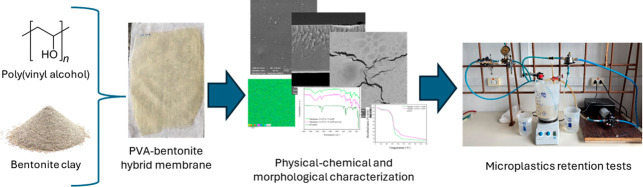

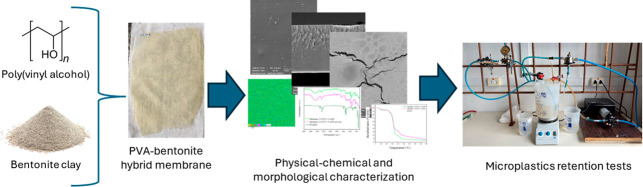

## 1 Introduction

The presence of microplastics (MPs) in water bodies has been recognized as one of the main contemporary environmental challenges, mainly due to their high persistence, resistance to degradation, and ability to act as vectors for associated contaminants, such as heavy metals and persistent organic compounds. Furthermore, their potential for bioaccumulation and biomagnification along the food chain is noteworthy, increasing ecological and human health risks.^1–5^ These particles, classified as emerging pollutants, have already been detected in surface water, groundwater, marine environments, and even in public water supply systems, highlighting their wide environmental dispersion and the limitations of conventional technologies in the efficient removal of these particulate fractions.^6–8^

Water Treatment Plants (WTPs), although highly efficient at removing turbidity, suspended solids, and dissolved organic matter, were not originally designed to retain polymeric particles at the micro- and nanoscale. Recent studies demonstrate that a significant fraction of PMs can bypass conventional coagulation, flocculation, sedimentation, and rapid filtration stages, especially when small or exhibiting colloidal behavior.^9–11^ Removal efficiency also depends on particles’ morphology, density, and chemical composition, further complicating the process.^12,13^

In this scenario, membrane separation processes stand out as a promising alternative due to their high selectivity and the potential for physical retention via size exclusion, interception, and the formation of a surface layer associated with concentration polarization and particulate fouling.^14^ Technologies such as microfiltration and ultrafiltration have demonstrated significant removal of micro and nanoplastics. However, challenges related to hydraulic compaction, fouling, and structural changes in the polymer matrix during operation persist.^14,15^

Regarding the materials used, poly­(vinyl alcohol) (PVA) stands out for its hydrophilicity, film-forming capacity, ease of cross-linking, and stability in aqueous media. It is being widely investigated for membrane applications.^16–18^ Polypropylene acetate (PVA) has been widely used in the development of polymeric membranes due to its favorable properties, such as biocompatibility, biodegradability, nontoxicity, and high film-forming capacity. Furthermore, it exhibits good chemical resistance and a high density of hydroxyl groups, which allow for structural modification of the matrix through processes such as cross-linking and incorporation of inorganic phases. These characteristics make PVA a promising material for water treatment applications, especially in the context of developing sustainable membranes. The incorporation of nanoclays, such as bentonite, contributes to the improvement of mechanical and thermal properties, in addition to modifying the internal morphology of the matrix through interactions between the polymer chains and the clay lamellae. In addition, it has been explored as a strategy for modulating the structural and functional properties of polymeric membranes. Due to its lamellar structure, high surface area, and ability to interact with polymeric matrices, bentonite can act as a reinforcing phase, influencing pore distribution, structural stability, and transport behavior of membranes. Furthermore, the presence of the inorganic phase can promote reorganizations in the polymeric matrix, affecting properties such as hydrophobicity, mechanical strength, and permeability, which directly impacts performance in separation processes.^16,19,20^ These polymer–clay nanocomposites can increase structural tortuosity and act as an additional barrier to particle transport, favoring predominantly physical retention mechanisms. It is worth noting that despite the widespread use of PVA membranes and the incorporation of nanoclays, there is still a gap in the mechanistic understanding of how the nanoparticle content simultaneously influences the structural organization of the matrix, the transport properties, and the retention efficiency of microplastics.^21,22^

Despite the advances, most studies focus on overall retention efficiency, with less depth in understanding the structural mechanisms involved and in evaluating the matrix’s stability after the filtration process. Differentiating surface retention associated with fouling layer formation from possible internal entrapment in the porous structure is fundamental to understanding the dynamics of the process, the evolution of hydraulic resistance, and the operational behavior of the system.^14,15^

Therefore, this study aims to evaluate the retention efficiency of polystyrene (PS) micromaterials in green PVA membranes additivated with ArNPben, as well as to elucidate the retention mechanism, and analyze the material’s structural stability after use. To this end, morphological (SEM-FEG), chemical (EDS and FTIR-ATR), thermal (TGA/DTG and DSC), and structural analyses were integrated, allowing for the correlation of removal performance, changes in the internal organization of the matrix, and possible effects of compaction or intermolecular reorganization. The approach adopted seeks to determine whether retention occurs predominantly through the formation of a surface layer or through relevant modifications in the nanocomposite’s internal structure, thereby advancing the development of sustainable membranes for water treatment.

Despite advancements in the use of polymeric membranes for microplastic removal, there is still a gap in understanding the mechanisms involved in the retention process, especially regarding the relative contribution between microplastic removal, fouling layer formation, and membrane structural changes during filtration. Furthermore, the influence of nanoparticle incorporation on fouling dynamics and membrane operational performance is not yet fully elucidated. In this context, this work stands out by investigating the performance of green PVA-based membranes, cross-linked with citric acid and reinforced with ArNPben, focusing on microplastic removal and structural modifications associated with the filtration process. The approach adopted integrates pre- and postuse morphological, spectroscopic, and thermal analyses, allowing for an indirect evaluation of fouling layer formation and its influence on system behavior.

The main innovation of the study lies in the correlation between the incorporation of ArNPben, the structural reorganization of the membrane, and the observed effects on PM retention and fouling formation, demonstrating the existence of an optimal composition that balances separation performance and structural stability. Additionally, the work contributes by demonstrating high retention efficiency along with evidence of surface particle deposition, offering an integrated view of the system’s performance and operational limitations. Thus, the study advances the understanding of the phenomena involved in PM filtration by hybrid polymeric membranes, with emphasis on the interaction between retention and fouling, contributing to the development of more efficient materials for water treatment applications.

## 2 Materials and Methods

### 2.1 Membrane Preparation

The methodology adopted in this study was structured to quantify microplastic retention efficiency, elucidate the structural mechanism underlying the process, and evaluate the stability of the polymer matrix after use. To this end, steps were integrated involving controlled preparation of the polystyrene (PS) suspension, transmembrane pressure filtration tests, and comparative characterizations performed before and after the retention process. The methodological approach was designed to ensure experimental reproducibility, preservation of postuse morphology, and integrated interpretation of results, thereby allowing simultaneous evaluation of removal performance and the material’s structural integrity.

For the production of the membranes, PVA (degree of hydrolysis greater than 99%, with a molar mass of 85–124 kDa) was acquired from Sigma-Aldrich; anhydrous citric acid PA (2-hydroxy-1,2,3-propanetricarboxylic acid), from Dinâmica Química Contemporânea; glycerol PA (propan-1,2,3-triol), from Vetec Química Fina; ArNPben (particle size less than or equal to 25 μm) were acquired from Sigma-Aldrich; PS pellets and recycled polystyrene (PSr) were donated by the company GramPlast Indústria e Comércio Eireli, and subsequently ground. The grinding took place in a Retsch Emax high-energy mill in 10 min batches at 1000 rpm, with the temperature controlled between 20 and 30 °C. The titanium spheres used in the grinding process have a unit mass of 0.208 and 0.494 g. The batches were carried out with 50 g of PS polymer each.

In a preliminary step to the aforementioned tests, the pure membranes (produced without additives) were subjected to the same characterization tests presented in this work, to verify at which concentration (6% PVA (w/v), 8% PVA (w/v) or 10% PVA (w/v)) would present the best structural assembly, considering the use of citric acid (10% w/w PVA) and glycerol (4% w/v) as cross-linking and malleabilizing agents, respectively. The best results were observed for the 6% PVA (w/v) membrane, which was determined to be ideal among the proposed concentrations to proceed to the additive stage. New solutions were prepared with a concentration of 6% PVA (w/v) and supplemented with 1% (mass of ArNPben nanoclay/mass of PVA polymer), 3% (w/w PVA) and 5% (w/w PVA), where it was possible to determine that the membrane with the best performance, among the proposed membranes, was the 6% PVA (w/v) membrane supplemented with 1% (w/w PVA).

The membranes were prepared using the solvent evaporation phase inversion method, utilizing PVA as the polymeric matrix. Aqueous PVA solutions were prepared at concentrations of 6, 8, and 10% (w/v), under stirring and heating until complete dissolution. Glycerol was added as a plasticizer at a ratio of 4% (v/v) relative to the solvent volume. Citric acid was used as a cross-linking agent at a ratio of 10% (w/w) relative to the PVA mass, being incorporated into the polymeric solution while still in the liquid phase. For the membranes with added additives, ArNPben was incorporated into the polymeric solution at concentrations of 1, 3, and 5% (w/w) relative to the PVA, being previously dispersed to ensure better homogeneity of the system.

The solutions obtained were poured onto flat surfaces to form the films, followed by controlled evaporation of the solvent under ambient conditions. After the membranes were formed, cross-linking of the polymer matrix was induced for 24 h at room temperature (25 °C) and finalized in an oven at 110 °C for 3 h, promoted by the presence of citric acid, resulting in the formation of a three-dimensional network stabilized by intermolecular interactions.

The presence of citric acid acted as a cross-linking agent for the PVA matrix, promoting the formation of intermolecular bonds between the polymer chains, which directly contributes to the structural stability of the membranes in aqueous medium. Although the degree of cross-linking and the gel content were not quantitatively determined, indirect evidence of the formation of the cross-linked network was observed through swelling and mass loss tests in water, which indicated good structural integrity of the membranes. Additionally, morphological analyses showed dense and continuous structures, compatible with cross-linked polymeric systems. These results indicated that cross-linking was sufficient to reduce the solubility of PVA in water, allowing the application of the membranes in filtration processes without compromising structural integrity.

### 2.2 Preparation of Microplastics

Polystyrene (PS) particles were obtained in two configurations: pristine pellets and recycled, pigmented white pellets. The material was previously sterilized and handled under controlled conditions to avoid microbiological interference or surface contamination that could compromise subsequent analyses. The choice of PS is justified by the ease of spectroscopic identification via FTIR-ATR, which allows distinction from poly­(vinyl alcohol) (PVA) through characteristic bands associated with aromatic rings. On the other hand, PS exhibits typical absorptions attributed to aromatic C–H vibrations (≈700–760 and 3025–3080 cm^–1^) and bonds associated with aromaticity (≈2850–2950 cm^–1^), while PVA displays bands related to O–H stretching (≈3200–3550 cm^–1^) and C–O stretching (≈1080–1140 cm^–1^), allowing for clear structural differentiation between matrix and particle during chemical analyses. The characteristic bands described are shown in Table 1.

**Table 1:** Infrared Reflectance Spectrum and Differences between PVA and PS^a^

spectral region (cm^–1^)	PS (polystyrene)	PVA (poly(vinyl alcohol))	main differences
3200–3550	C–H aromatic	absent	exclusive to PS-Aromaticity
3025–3080	C–H aliphatic	C–H aliphatic	both have it, but PVA is only aliphatic.
2850–2950	C=C aromatic	absent	PS exclusive
1600–1490	absent	O–H stretch	exclusive to PVA
1080–1140	absent	C–O stretch	exclusive to PVA
700–760	C–H aromatic out of plane	absent	PS exclusive

a Source: Authors (2026).

To obtain fractions within the characteristic size range of MPs (1 μm to 5 mm), the pellets were ground in a high-energy mill (Retsch Emax). The process was carried out in ten batches of 30 g, totaling 300 g of ground material, to ensure enough for particle-size standardization and suspension preparation. The solution containing polystyrene (PS) MPs was prepared from standard particles with a nominal diameter of 2.7–299.0 μm, selected because they span a size range compatible with particles frequently identified in ambient aqueous matrices. Dispersion was carried out in distilled water under controlled agitation to minimize agglomeration and ensure homogeneous particle distribution in the liquid phase.

Before the retention tests, the suspension was homogenized to ensure dispersion stability throughout the experimental period. This procedure was fundamental in reducing interferences caused by sedimentation or particle aggregation, allowing the retention process to be predominantly governed by the membrane’s structural characteristics rather than by suspension instabilities. The concentration used (2.0 g·L^–1^) was determined based on performance verification in a system loaded with the pollutant, ensuring experimental reproducibility and comparability of results.

### 2.3 Microplastic Retention Test

Retention tests were performed in a pressure-permeation system operating in a tangential-flow (dead-end) regime, using a filtration cell coupled to a pressurized reservoir and a pressure gauge to control the transmembrane pressure. The permeation system used in the test is shown in Figure 1. The previously characterized membranes were fixed in the cell, ensuring adequate sealing and a constant effective area between tests. The test was carried out at a constant pressure of 3 bar, due to a change of approximately ± 0.5 bar above this. This was associated with the presence of polymeric particles in the solution, which, when passing through the system’s pump, caused changes that prevented the test from being carried out at higher pressures. However, at the chosen pressure, permeation was satisfactorily performed without altering the permeate stream. The system was operated with distilled water as the continuous phase, under standardized conditions, to ensure that variations in retention efficiency were attributable to the structural properties of the membranes. The choice of the dead-end regime was intentional, as it favors the formation of concentration polarization, allowing for subsequent morphological analysis of the surface after use.

**Figure 1:**
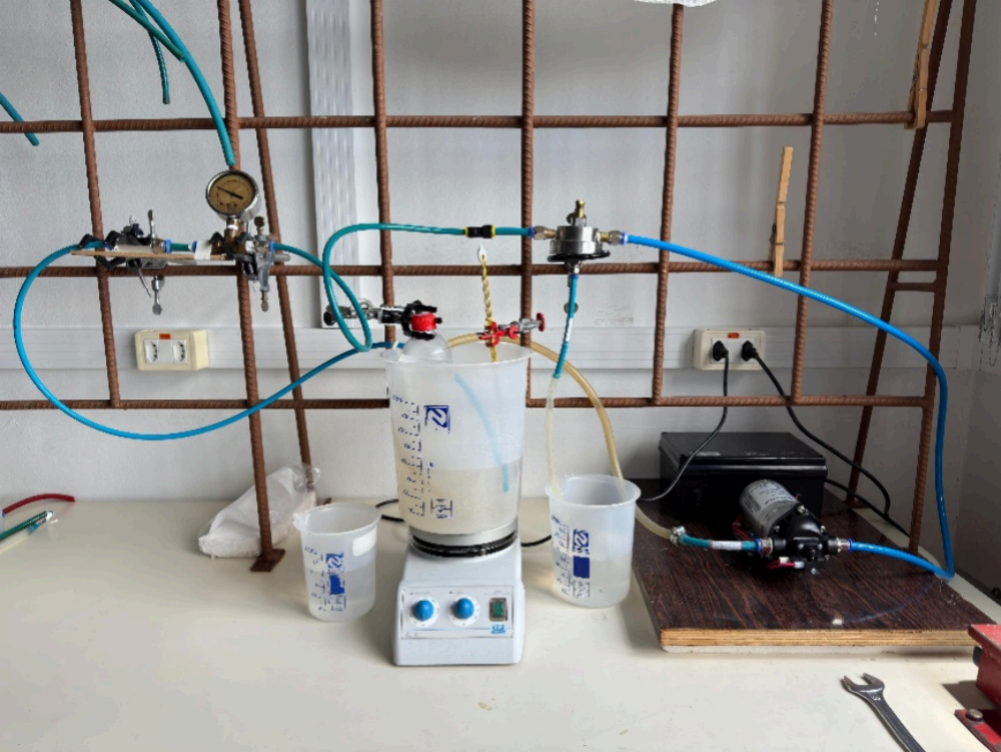
Permeation system with agitation used in the experiments. Source: Authors (2026).

The analysis was concluded after 1 L of solution was collected in the permeate stream, which was stored at a controlled temperature in a freezer (−18 °C) to prevent colonization by microorganisms until the lyophilization tests were performed to determine the polymer mass in the permeate stream.

The samples were subjected to lyophilization, involving prior freezing followed by sublimation drying under low pressure, to ensure that no alteration occurred in the structure of the retentates or permeates, which allowed the determination of particle retention. Lyophilization was conducted under reduced pressure (0.5 bar) at 110 °C, allowing the solid phase to sublimate directly into vapor. The use of lyophilization ensures that the structure of the retentates or permeates is not altered, allowing particle retention to be determined.

### 2.4 Description of Characterization Tests and Comparison between Samples

Morphological characterization was performed by field emission scanning electron microscopy using a TESCAN MIRA3 electron microscope. Before analysis, the samples were subjected to cryogenic fracture in liquid nitrogen, placed on a stub with carbon tape, and coated with a thin layer of gold for 3 min by sputtering, thereby metallizing them to enable image acquisition and analysis. This test allows high-resolution micrographs to be observed and provides insights into the distance between the highest and deepest points of the membrane surface and its cross-section (topography).^23^ This applies to membranes both before filtration and after the retention test and lyophilization process.

The preuse analysis aimed to establish a structural reference for the polymer matrix. The postuse analysis was conducted to identify possible morphological changes resulting from filtration, including the formation of a surface layer, partial pore obstruction, or eventual collapse of the internal structure. The comparison between pre- and postuse micrographs allowed us to assess whether retention occurred predominantly on the membrane surface or whether particles were entrapped within the membrane.

Energy-dispersive X-ray spectroscopy (EDS), coupled with SEM, was used to identify characteristic elements associated with PS particles on the membrane surface after the retention test. The analysis was performed on specific regions previously identified in the micrographs, enabling correlation between morphology and chemical composition. The detection of a characteristic carbon signal in surface regions served as an indicator of polymeric particle retention, reinforcing the interpretation of the surface retention mechanism. The comparison of membranes before and after use confirmed the absence of intrinsic chemical alterations to the PVA matrix caused by the filtration process.

Fourier transform infrared spectroscopy (FTIR-ATR) was performed on the membranes before and after the retention process, to verify possible alterations in the characteristic bands of PVA and to identify any presence of signals associated with polystyrene. Membranes without additives, with additives, and with additives added after use for permeation were tested using a Thermo Scientific Nicolet IS10 spectrophotometer (USA) to verify the composition of the materials used. The analysis was performed in attenuated total reflection (ATR) mode, averaging 32 scans over 4000–400 cm^–1^ with a resolution of 4.0 cm^–1^.

Comparative analysis allowed us to assess whether the hydrogen-bonding network of the polymer matrix was modified after filtration and to investigate possible chemical interactions between the retained particles and the membrane surface. The preservation of characteristic PVA bands, along with the identification of PS-related bands on the surface, indicated predominantly physical retention.

TGA/DTG and DSC analyses were performed on the membranes before and after the retention test, allowing us to assess whether the filtration process altered the thermal stability or the matrix’s structural organization. The thermal stability of the obtained PVA membranes was evaluated using a SHIMADZU TGA-50 thermogravimetric balance. Approximately 10 mg of the sample was analyzed in an inert nitrogen atmosphere at a flow rate of 50 mL·min^–1^, using a platinum sample holder heated from 25 to 900 °C at a heating rate of 10 °C·min^–1^. DTG data were generated by the equipment’s own software using the TGA data. DSC analysis of the obtained PVA membranes was performed using a SHIMADZU DSC-60 instrument, with a temperature range of 25–250 °C, a heating rate of 10 °C·min^–1^, and a nitrogen gas flow rate of 50 mL·min^–1^. The mass used for this analysis is approximately 10 mg. The glass transition temperature (*T*_g_), melting temperature (*T*_m_), and enthalpy of fusion (Δ*H*_f_) results were obtained from the curves generated during the first heating to evaluate the effects of processing on the membrane, while the crystallization temperature (*T*_c_) was extracted from the curve generated during the cooling step.

Comparing thermogravimetric profiles and enthalpies of fusion (Δ*H*) allowed identification of changes associated with retained particles or structural reorganization induced by the hydraulic pressure process. The maintenance of the matrix’s original thermal profile was interpreted as indicative of postuse structural stability, while significant alterations could suggest deeper modifications in the supramolecular organization.

The experiment followed a completely randomized design, with the analysis factors being the concentration of PVA in the polymer solutions, the amount of citric acid additive used, the preparation of solutions with MP particles, and the retention of MP particles in the test performed. All tests were performed in triplicate. The data were subjected to a Student’s *t* test at a 5% significance level, using JASP statistical software (Netherlands).

## 3 Results and Discussion

### 3.1 Pre- and Postuse Morphological Analysis

Morphological analysis using scanning electron microscopy is a central element for understanding the retention mechanism. The micrographs obtained before the filtration test confirm the previously discussed structural organization, characterized by a continuous matrix and compact cross-section, without relevant structural discontinuities. The micrographs obtained at this stage are shown in Figures 2 and 3.

**Figure 2:**
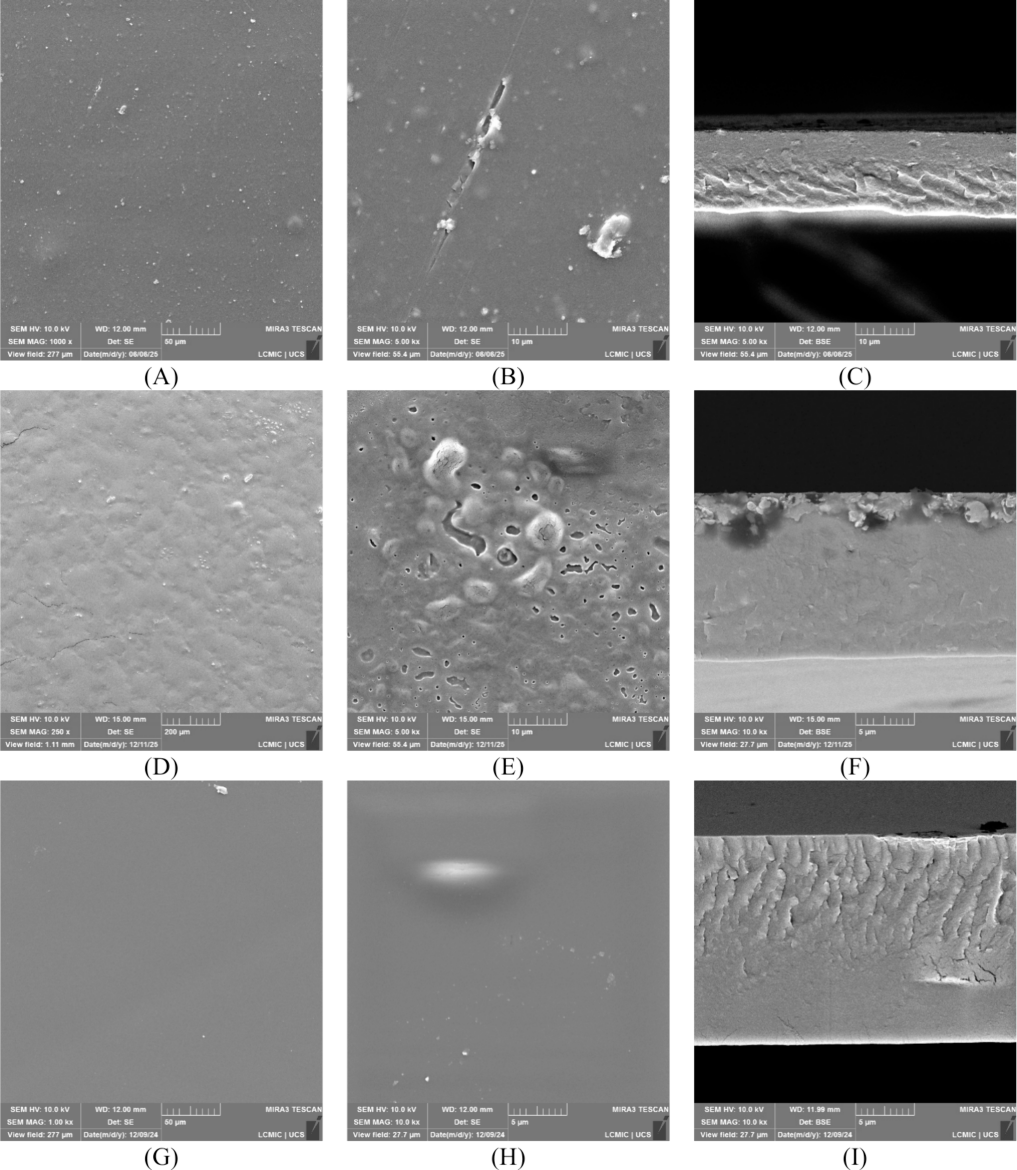
Micrographs obtained for the membranes and concentrations of (A) 6% PVA (w/w) 1% ArNPben (w/v)—preuse top view, (B) 6% PVA (w/w) 1% ArNPben (w/v)—approximate preuse top view, (C) 6% PVA (w/w) 1% ArNPben (w/v)—preuse cross-section, (D) 6% PVA (w/w) 1% ArNPben (w/v)—postuse top view, (E) 6% PVA (w/w) 1% ArNPben (w/v)—approximate postuse top view, (F) 6% PVA (w/w) 1% ArNPben (w/v)—postuse cross-section, (G) 6% PVA (w/w) without additives—preuse top view, (H) 6% PVA (w/w) without additives. Additives—approximate top view before use, (I) 6% PVA (m/m) without additives—cross section before use. Source: Authors (2026).

**Figure 3:**
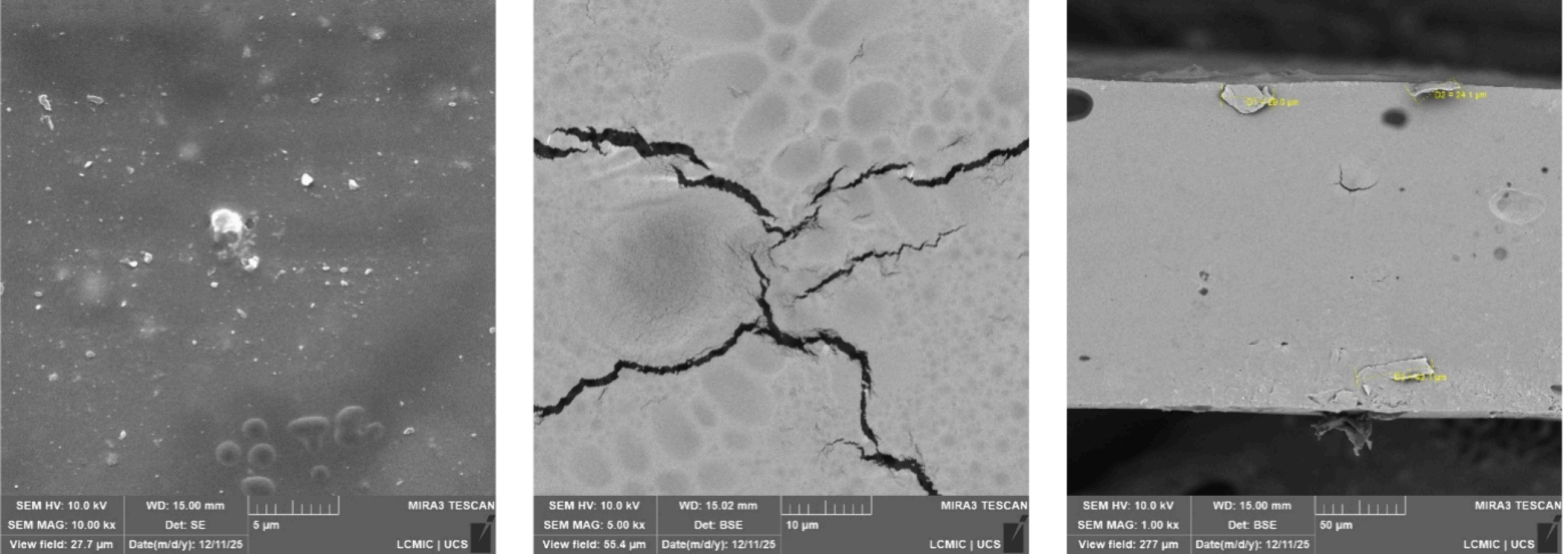
Micrographs obtained for the membranes and concentrations of 6% PVA (w/w) 1% ArNPben (w/v)—top view after use—notes of discontinuities and the appearance of fouling. Source: Authors (2026).

The cross sections of the postuse membranes showed no significant internal structural changes compared to the original micrographs. No evidence of deep obstruction, fissure formation, or matrix structural reorganization was observed, indicating that deposition occurred predominantly on the outer surface. This behavior is characteristic of systems in which concentration polarization formation and surface particulate fouling govern the retention mechanism, especially under perpendicular flow regimes.^14,24,25^

The absence of internal structural modification reinforces the hypothesis that retention is not due to intraporous entrapment, but rather to initial physical exclusion, associated with the dense nature of the cross-linked PVA polymer matrix, followed by progressive surface deposition. PVA-based membranes, especially when modified with nanoclays, exhibit structural organization with increased tortuosity and reduced permeability to solid particles, favoring predominantly surface retention mechanisms.^16,19,21^

The structural stability observed after the filtration process is also consistent with studies indicating that PVA/clay nanocomposites exhibit adequate mechanical and thermal integrity, maintaining matrix organization even under hydraulic flow and particle interactions.^16,17,20^ This behavior suggests that the increase in hydraulic resistance during the test is mainly due to the formation of a surface layer rather than to permanent internal changes in the polymer matrix.

EDS analysis was used as a qualitative tool to evaluate the distribution of elements on the membrane surface, especially those associated with the inorganic phase (Si, Al, and Mg from bentonite). After use, surface changes compatible with the deposition of material on the membrane surface were observed. However, it is important to note that EDS has limitations in distinguishing between carbon-rich materials, such as PVA and PS, and it is not possible to unequivocally confirm the presence of PS based solely on carbon mapping. Therefore, EDS results should be interpreted as complementary evidence. The use of SEM-EDS as a complementary tool for confirming particulate deposition is widely described in the literature for evaluating fouling and identifying polymeric materials retained on membrane surfaces.^22,25^ These results corroborate the morphological interpretation and confirm that the layer formed is mainly composed of PS MPs, as illustrated in Figure 4.

**Figure 4:**
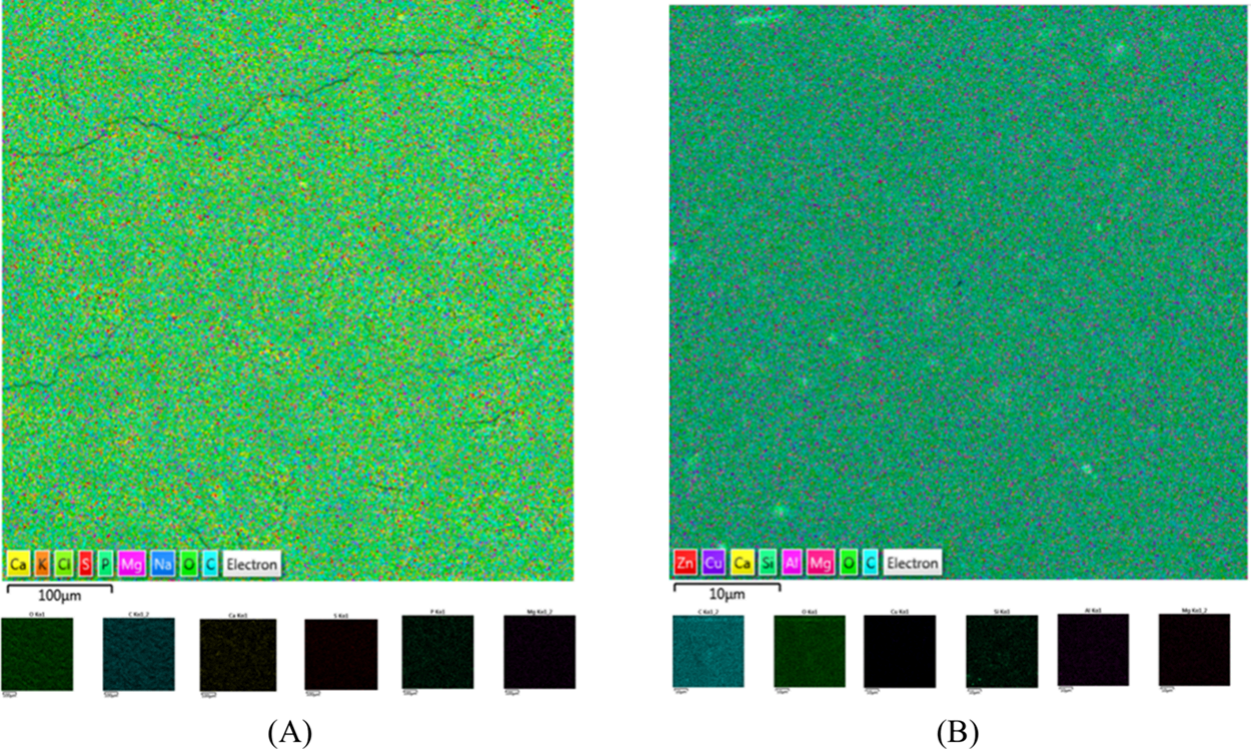
Energy-dispersive spectroscopy (EDS) images for the comparison of the surface before (A) and after (B) use of the additive membranes obtained. Source: Authors (2026).

Compared with the membranes before the test, the membranes after the test did not show any localized surface carbon concentration that could be attributed to an additional phase distinct from the polymeric matrix. The detected signal was homogeneous and compatible with the organic composition of PVA itself, with no evidence of specific surface accumulation. After the test, however, a predominance of the carbon signal was observed in the external region.

This behavior is directly associated with the physical exclusion retention mechanism, which may be one of the retention mechanisms presented in this work, in which particles with dimensions larger than the effective passage diameter are prevented from crossing the structural barrier and progressively accumulate on the membrane surface.^14,26^ In dense systems or those with high structural tortuosity, as observed in cross-linked PVA membranes modified with nanoclays, particulate transport is predominantly governed by initial surface blockage, followed by the formation of a deposited layer.^16,19,21^

Furthermore, the absence of a carbon signal distributed internally along the transverse fracture rules out the hypothesis of intraporous fouling or internal channel blockage, phenomena commonly associated with microporous or asymmetric membranes with interconnected pores.^25,27^ In the present case, the compact structural organization of the polymeric matrix, associated with the possible reduction of free volume by the incorporation of bentonite clay, acts as an additional barrier to particulate migration, favoring essentially superficial retention.^17,20^

The progressive formation of a deposited layer can also intensify polarization by concentration, increasing hydraulic resistance over time.^14,24^ However, since no relevant internal structural changes were identified, it can be inferred that the increase in resistance observed during the tests is mainly due to the additional resistance imposed by the MP’s surface layer, rather than to structural compaction of the matrix or collapse of the internal structure.

Thus, the results obtained by SEM-EDS corroborate the mechanism of superficial physical exclusion as the main pathway for the retention of PS MP’s, reinforcing that the observed performance stems from the structural barrier of the dense membrane, associated with progressive external deposition, without compromising the internal integrity of the polymeric hybrid matrix. MP retention was confirmed mainly by infrared spectroscopy (FTIR), in which characteristic bands associated with the aromatic ring of PS were observed after the filtration test, and using thermogravimetric analysis (TGA), which corroborated this interpretation, showing changes in the thermal degradation profile of the membranes after use. Therefore, the confirmation of MP retention was based on the integrated analysis of FTIR and TGA techniques, while EDS was used as a complementary tool for evaluating surface changes and elemental distribution.

### 3.2 Comparative Analysis Using FTIR-ATR

The FTIR-ATR spectra of the membranes and the pure polymer are shown in Figure 5, while the characteristic bands of each material are described in Table 2. The 6% PVA 1% ArNP membrane, before the filtration test, showed the typical profile of cross-linked PVA, with bands mainly associated with the O–H and C–O groups.

**Figure 5:**
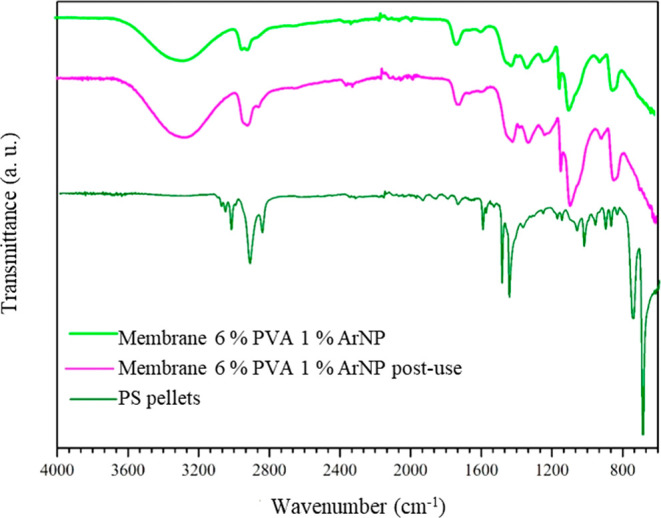
FTIR spectra of the 6% PVA 1% ArNP membrane before and after the MP retention test, compared to the FTIR spectrum of pure polystyrene (PS). Source: Authors (2026).

**Table 2:** Main Bands Observed in the FTIR Spectra for 6% PVA and 1% ArNPben Membranes before and after Use, Compared to the Spectrum of PS^a^

wavenumber (cm^–1^)				
6% PVA 1% ArNPben preuse	6% PVA 1% ArNPben postuse	PS pellets	chemical bond	deformation
3200–3500	3200–3500		OH PVA	axial stretching
	∼3020	∼3020	aromatic tea	axial stretching
2940	2940	2920–2850	C–H (−CH_2_−)	axial stretching
∼1650	∼1650		O–H (adsorbed water)	angular deformation
	∼1600	∼1600	aromatic C=C (benzene ring)	axial stretching
	∼1490	∼1490	C=C aromatic	axial stretching
1140–1080	1140–1080		C–O (PVA)	axial stretching
	∼750	∼750	aromatic C–H (outside the plane)	deformation outside the plan
	∼700	∼700	aromatic C–H (outside the plane)	deformation outside the plan

a Source: Authors (2026). Adapted from Coates;^29^ Mansur;^30^ Bergaya.^31^

The FTIR spectra of the membranes before and after use did not show significant differences, which may be attributed to the predominance of the characteristic bands of PVA, which tend to mask lower intensity contributions from PS. The broad bands associated with hydroxyl groups (3200–3500 cm^–1^) and C–O vibrations (1140–1080 cm^–1^) dominate the spectrum, making it difficult to unequivocally identify additional signals. Small variations can be observed in the region of ∼1600–1500 cm^–1^, associated with the vibrations of the aromatic ring of PS, but with low relative intensity. This behavior suggests that the amount of retained material, although present, may not be high enough to generate significant spectral changes in the FTIR. Therefore, FTIR analysis should be interpreted in a complementary manner, with confirmation of MP retention based on integrated analysis with other techniques, such as TGA and SEM, which show structural and thermal changes after membrane use.

The spectrum of the PS pellets exhibited the characteristic vibrational signature of the aromatic ring, with bands at approximately 1600–1490 cm^–1^ attributed to C=C stretching of the benzene ring, in addition to out-of-plane C–H aromatic deformations at ∼750 and ∼700 cm^–1^, and aromatic C–H vibrations at ∼3020 cm^–1^. This set constitutes a robust spectral pattern for identifying particulate matter after retention tests.^29,30^

Although bands associated with polystyrene were identified in the surface region, no relevant shifts or alterations in the characteristic structural bands of PVA were observed that would indicate the formation of new chemical bonds or a substantial supramolecular reorganization of the matrix. The bands assigned to the O–H and C–O groups remained at their positions and spectral profiles, demonstrating structural stability after the filtration test. Statistical analysis of the relative intensities of the main structural bands confirmed the absence of a significant difference (*p* > 0.05) between the pre- and postuse samples, reinforcing that the retention process did not promote chemical modifications in the polymeric matrix. These results indicate that the interaction between the membrane and the MPs occurs predominantly through physical mechanisms.

This behavior is consistent with Zhao,^28^ who highlights that, in the absence of specific chemical interaction, the retention of polymeric particles by hydrophilic membranes occurs mainly through physical exclusion and progressive surface deposition. Similarly, Baker^14^ and Greenlee^26^ report that, in systems where there is no chemical affinity between the solute and the matrix, removal efficiency is associated with the membrane’s structural barrier and the formation of a surface layer, rather than with incorporation or chemical reaction with the polymeric structure.

Furthermore, studies involving PVA membranes and polymer–clay nanocomposites demonstrate that, when the interaction with retained particles is predominantly physical, the matrix maintains its original structural organization, preserving chemical properties and stability after use.^16,19,20^ Thus, the spectroscopic results corroborate the morphological analyses and confirm that PS MPs are retained without compromising the polymeric matrix.

### 3.3 Comparative Thermal Analysis (TGA and DSC)

Although both samples exhibit similar behavior in the initial region (<120 °C), associated with the loss of physically adsorbed moisture, the postuse membrane showed a higher remaining mass fraction and a change in slope between 350–450 °C. This modification is consistent with the presence of retained polystyrene, whose typical thermal degradation range overlaps this range, indicating a distinct additional contribution from the original PVA matrix.^16,32^

Furthermore, an increase in final residue above 600 °C was observed in the postuse membrane, suggesting a combined effect of the inorganic phase (ArNPben) and carbonaceous residues from PS degradation. Together with the FTIR results, which qualitatively confirm the presence of aromatic structures, TGA, elucidated in Figure 6, provides complementary quantitative evidence of the physical retention of microplastics in the membrane.^16,33^

**Figure 6:**
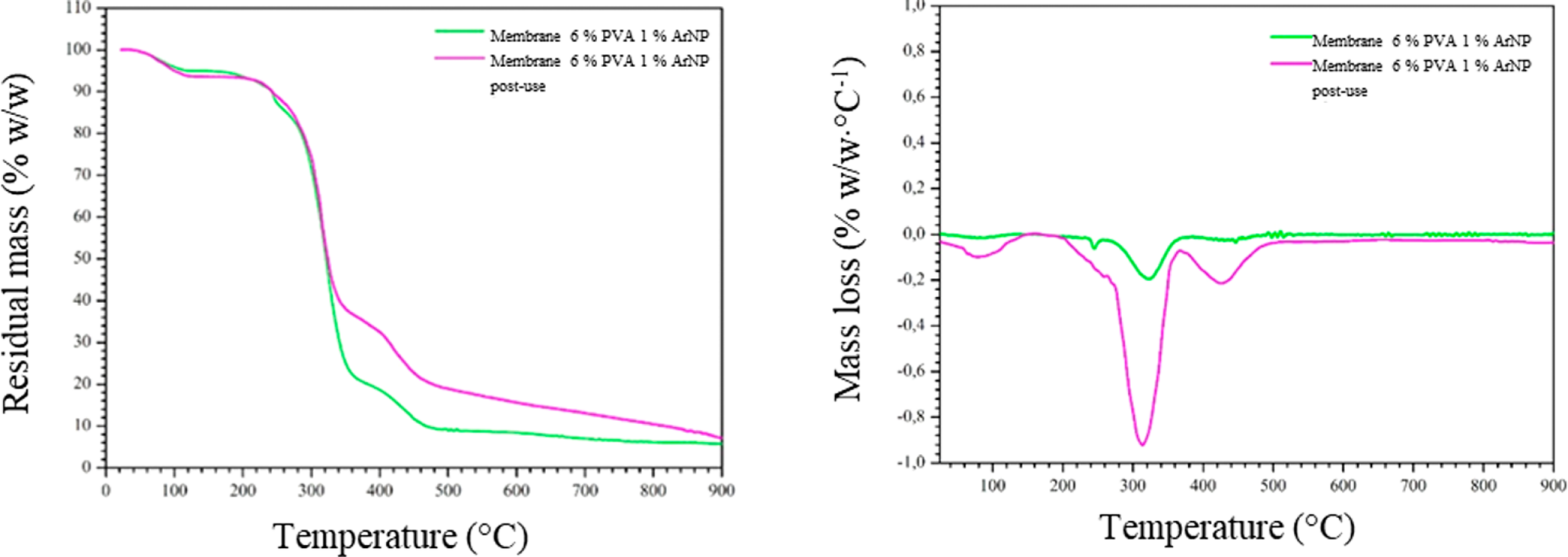
Thermogravimetric curves (A) and thermogravimetric derivatives (B) of the 6% PVA 1% ArNPben membranes before and after use in the MP retention test. Source: The Author (2026).

DTG analysis reinforces this interpretation, since, while the preuse sample shows a main peak between 280–320 °C, associated with structural dehydration and chain scission of PVA, the postuse membrane shows an intensification of this event and greater definition of a second peak between 380–450 °C, coinciding with the typical PS decomposition range.^16,32,33^ The increase in the area under the peaks indicates a higher overall rate of mass loss, evidencing effective incorporation of the microplastic into the matrix after the test.

Thus, DTG complements TGA by providing more conclusive evidence of retained PS, corroborating the spectroscopic and morphological results and reinforcing the physicochemical confirmation of surface retention and fouling formation on the membrane.

In the first DSC heating cycle (Figure 7A), the membrane before use exhibits a broad endothermic event between 70–110 °C, associated with the evaporation of physically adsorbed water and the structural relaxation of PVA. This behavior is characteristic of membranes prepared by casting, due to the polymer’s hygroscopic nature and the extensive hydrogen-bond network in the matrix. The endothermic valley indicates energy consumption related to the rupture of these intermolecular interactions and the initial reorganization of the polymer chains.^34–36^

**Figure 7:**
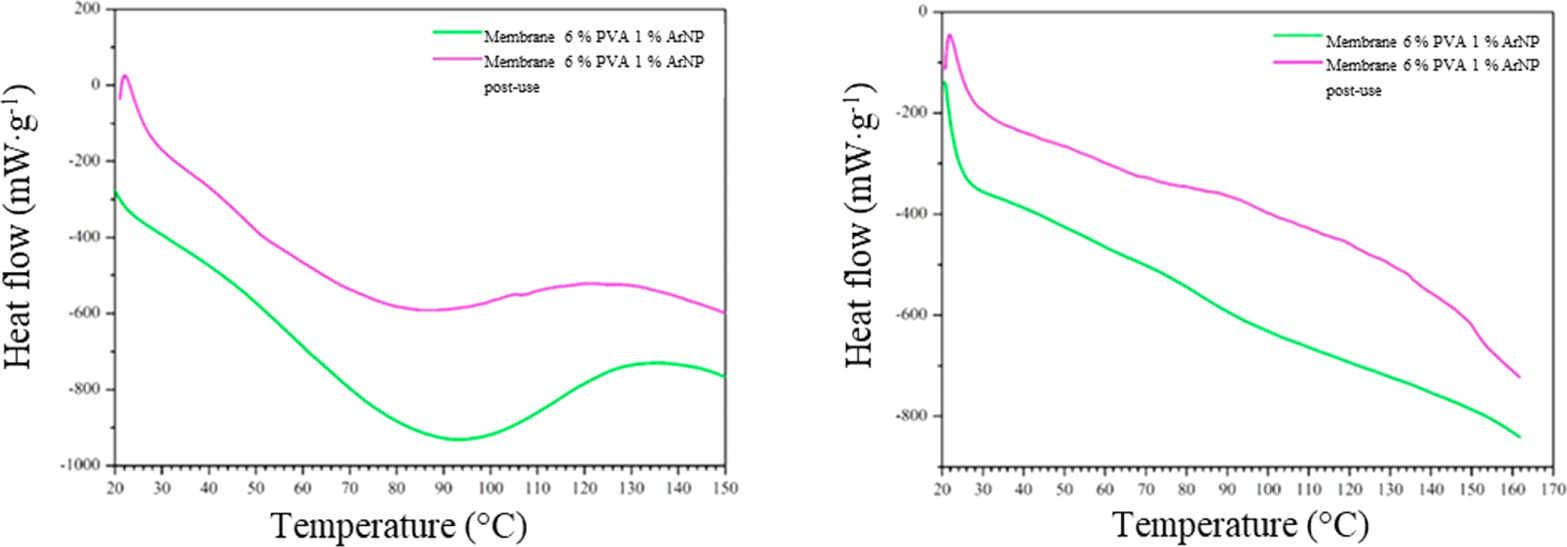
DSC curves for the first (A) and second (B) heating of the 6% PVA 1% ArNPben membranes before and after use in the MP retention test. Source: Authors (2026).

After use, a reduction in the intensity of the endothermic event and a change in the baseline across the temperature range are observed, suggesting a modification in the system’s energy balance. The presence of retained PS, which is hydrophobic, can reduce the water fraction associated with the matrix and, consequently, the enthalpy of evaporation, in addition to partially restricting the segmental mobility of PVA. This behavior indicates that the incorporation of microplastics not only contributes to mass variation, as evidenced by TGA/DTG, but also alters the membrane’s thermal dynamics and supramolecular organization.^36^

In the second DSC heating cycle (Figure 7B), a more stable thermal profile was observed, with the broad endothermic event associated with water evaporation disappearing. The membrane before use exhibits a relatively continuous baseline, with a slight variation in the estimated glass transition temperature (*T*_g_) of the partially cross-linked PVA (≈70–90 °C), as evidenced by a subtle change in heat flux. This behavior indicates that, after moisture removal and structural stabilization, the thermal response is predominantly governed by the segmental mobility of the polymeric matrix.^34^

In the postuse membrane, a change in the baseline slope was observed between approximately 60–120 °C, suggesting a modification in the apparent heat capacity of the system. The presence of retained polystyrene, acting as a rigid dispersed phase, partially restricts the segmental mobility of PVA, promoting redistribution of amorphous regions and a local increase in stiffness. Considering that PS has a higher *T*_g_ than plasticized PVA, its incorporation by fouling influences the overall molecular dynamics, as reflected in the observed calorimetric profile.^35,36^

Additionally, the absence of new defined endothermic peaks indicates that no additional crystalline phase formed and that no chemical reaction was observed during use, in agreement with the FTIR-ATR and TGA results previously discussed.^34,36^ Thus, the detected changes are predominantly associated with the physical modification of the structure and the thermal contribution of the incorporated PS, confirming that the microplastic is superficially integrated into the matrix and measurably affects the system’s thermal response.

### 3.4 Microplastic Retention Test

Particle retention tests were conducted in triplicate at a constant transmembrane pressure of 3.0 bar to evaluate the operational stability of the system and the reproducibility of the selected membrane.^14,26^ Retention efficiency was determined from the gravimetric quantification of the masses recovered in the permeate stream after the lyophilization process, a procedure adopted to ensure complete removal of the liquid phase and allow for the precise determination of the residual particle mass.^33^

The initial mass of MPs added to the solution ranged from 2.0004 to 2.0175 g, while the masses recovered in the permeate stream were significantly lower, resulting in removal percentages of 99.89, 99.82, and 99.64%, respectively. The average value obtained was 99.78 ± 0.13%, demonstrating high retention efficiency. The low dispersion among the triplicates, expressed by a standard deviation of 0.13%, confirms the operational consistency of the system under constant pressure and the structural uniformity of the membrane throughout the tests.^16,20^

It should be noted that a complete mass balance of the permeation system was not possible, as some particles remained encrusted on the equipment’s internal surfaces after the process was completed, requiring successive washes to remove them completely. Even so, this limitation did not compromise the determination of membrane efficiency, as evidenced by the comparison between the initial mass of the feed solution and the final mass recovered in the permeate after lyophilization.^14,26^

The near-100% efficiency suggests that the observed retention can be attributed to a combination of mechanisms, including size exclusion, fouling layer formation, and physicochemical interactions between the particles and the membrane surface, since the polystyrene particles used are larger than the effective pore size of the PVA matrix. Furthermore, during permeation, the progressive formation of a surface layer of particles may have contributed to an increase in local hydraulic resistance, reinforcing the blockage and reducing the residual passage of particulate matter. Mass losses resulting from interactions between the permeation system tubes and the PS particles were considered. After each permeation test, the piping was thoroughly cleaned and washed to capture any particles that may have adhered to the system, allowing them to be quantified along with those retained by the membrane. The results indicate that the improvement in MP retention is not only associated with the size exclusion effect, but also with the structural reorganization of the polymer matrix induced by the presence of ArNPben, which alters the pore distribution and increases resistance to transport.^14,24,25^

The small variations observed between the tests may be due to partial particle adsorption on the system’s internal surfaces or to discrete concentration polarization effects at the membrane-solution interface. This phenomenon, characterized by the accumulation of particles in the region adjacent to the membrane surface, can cause punctual variations in transmembrane flux by locally altering the hydrodynamic regime and the total transport resistance. In systems operating under constant pressure, such as the present one, polarization tends to intensify until it reaches a quasi-steady state, in which convective transport toward the membrane balances with the reverse diffusion of particles into the liquid medium.^37,38^

This balance explains the maintenance of high removal efficiency throughout the test, even in the presence of a possible surface layer of filter cake. The low dispersion between triplicates reinforces the experimental reliability of the results obtained and demonstrates that the system operated stably, without structural compromise of the membrane during the filtration process.^37,38^

## 4 Conclusions

In this work, it was observed that the controlled incorporation of bentonite promotes significant changes in the membrane structure, making it possible to identify an optimal additive concentration that maximizes performance by balancing structural resistance and transport capacity. The results obtained allow us to conclude that the retention of polystyrene MPs by green PVA membranes with bentonite additives occurs mainly through physical exclusion, accompanied by the progressive formation of a surface layer, without evidence of intraporous entrapment or of a relevant structural reorganization of the matrix. The high average efficiency of 99.78 ± 0.13% confirms the system’s operational stability and reproducibility under constant pressure. Integrated SEM-EDS, FTIR-ATR, and TGA/DTG/DSC analyses demonstrated surface deposition of PS, absence of chemical interaction, and preservation of the thermostructural integrity of the nanocomposite after use. Thus, the proposed objective was achieved by elucidating the structural retention mechanism and demonstrating that the observed performance is directly related to the membrane’s dense barrier, without compromising its internal organization, thereby reinforcing its potential for sustainable application in the treatment of water containing microplastics. In future studies, the inclusion of flow curves over time would enrich the understanding of the mechanisms involved in fouling layer formation, concentration polarization, and hydraulic resistance. Furthermore, pore size/MWCO characterization could be verified to corroborate the results obtained on PM retention and elucidate the efficiency of removal through its actual mechanism.
